# The effect of halothane and pentobarbital sodium on brain ependymal cilia

**DOI:** 10.1186/2046-2530-1-12

**Published:** 2012-07-06

**Authors:** Chris O’Callaghan, Kulvinder Sikand

**Affiliations:** 1 Department of Infection, Immunity and Inflammation, Division of Child Health, University of Leicester, Leicester, LE2 7LX, England, UK; 2 Department of Respiratory Medicine, Portex Unit, Institute of Child Health, University College London (UCL) and Great Ormond Street Hospital, 30 Guilford Street, London, WC1N 1EH, England, UK

**Keywords:** Cilia, Ciliary beat frequency, Ependyma, Ependymal cilia, Cerebrospinal fluid, CSF, CSF flow, Halothane, Volatile anesthetics, Brain cilia, Sodium pentobarbitone

## Abstract

**Background:**

The effect of anesthetic agents on ependymal ciliary function is unknown. The aim of this study was to determine the effect of halothane and pentobarbital sodium on brain ependymal ciliary function.

**Methods:**

We used an *ex vivo* rat brain slice model to measure ependymal ciliary beat frequency by high speed video photography at 37°C.

**Results:**

Exposure to halothane caused a significant reduction in ciliary beat frequency of 2 % (*P* = 0.006), 15.5 % (*P* < 0.001), and 21.5 % (*P* < 0.001) for halothane concentrations of 1.8 %, 3.4 % and 4.4 %, respectively, compared to controls. Following a one-hour wash-out period, there was no significant difference between control samples and cilia that had been exposed to 1.8 % (*P* = 0.5) and 3.4 % (*P* = 0.3) halothane. The beat frequency of cilia exposed to 4.4 % halothane had increased following the wash-out period but cilia were still beating significantly more slowly than cilia from the control group (*P* = <0.001).

Pentobarbitone at concentrations of 25 and 50 μg/ml had no effect on ciliary beat frequency compared to controls (*P* = 0.6 and 0.4 respectively). A significant (*P* = 0.002) decrease in ciliary beat frequency was seen following incubation with a pentobarbitone concentration of 250 μg/ml (mean (SD) frequency, 24(8) Hz compared to controls, 38(9) Hz).

**Conclusions:**

Halothane reversibly inhibits the rate at which ependymal cilia beat. Pentobarbitone has no effect on ciliary activity at levels used for anesthesia. It is unclear whether the slowing of ependymal ciliary by halothane is responsible for some of the secondary central nervous system effects of volatile anesthetic agents.

## Background

Ciliated ependymal cells line the ventricular system of the brain [1]. They have approximately 40 rapidly beating cilia that move the cerebrospinal fluid (CSF) immediately adjacent to the ventricular surface [2,3]. The function of ependymal cilia is still unclear; however, there is good evidence that abnormal ependymal ciliary function during development is associated with the development of hydrocephalus [4-10]. It has been shown that toxic insults, such as exposure to bacteria and bacterial toxins, can adversely affect ependymal ciliary function, both *in vitro*[11-13] and *in vivo*.

We were eager to study the effect of halothane, an inhalational anesthetic and pentobarbitone, a barbiturate, on ependymal ciliary function for two reasons. First, it would provide further information on the central nervous system effects of these agents in patients. For example, halothane exposure is associated with a rise in intracranial pressure [14]. Second, halothane and pentobarbitone are commonly used as anesthetic agents for *in vivo* brain and CSF fluid studies [15-22]. It is possible that interpretation of such studies may benefit from knowledge of the effect of these drugs on ependymal ciliary function. Our concerns were based on evidence that a number of anesthetic agents have been shown to cause significant slowing of respiratory ciliary beat frequency [23-25].

The depressant effects of anesthetic agents on the cilia of the ciliated protozoan, *Tetrahymena pyriformis,* were described by Nunn [26]. Inhalational anesthetic agents have been shown to depress mucociliary transport in the respiratory tract *in vivo* in animals and in humans [27,28]. Manawadu *et al*. investigated the effects of halothane on ferret tracheal cilia by noting the presence or absence of ciliary activity at different sites and reported a reduction in the number of functioning cilia after prolonged exposure [29]. Lee and Park found the activity of cilia from rabbit tracheal specimens was reduced by halothane and enflurane [30]. Gyi *et al*. [24] showed that human respiratory cilia beat frequency may be reversibly inhibited by halothane and Raphael and colleagues found similar levels of ciliary depression following the exposure of respiratory cilia to isofluorane and enflurane [25]. No investigations have been performed on the effect of anesthetics on brain ependymal ciliary beat frequency. Although ciliary structure is similar in different species, ciliary function and mechanisms of ciliary control are not uniform [31,32], making it difficult to extrapolate results from tissue to tissue or from animal to animal.

The aim of this study was, therefore, to investigate the effect of various concentrations of halothane and pentobarbitone on brain ependymal ciliary function.

## Methods

### Sample preparation

We have previously described the methods used for sample preparation and beat frequency analysis [12,33]. Brain slices were prepared from the floor of the fourth ventricle of Wistar rats (9 to 15 days of age) immediately after sacrifice and mounted in a well containing 4 ml of medium 199 with Earle’s salts (pH 7.4: plus penicillin 50 u/ml and streptomycin 50 μg/ml) and kept at 4°C until the study began. For the experiment, the well was placed in a purpose built environmental chamber which was thermostatically controlled to keep the fluid surrounding the ependymal sample at 37°C. The chamber was humidified to 80 % to prevent evaporation from the well during the three-hour study period. Ciliary movement was observed using a x50 lens [34]. Studies were conducted within eight hours of sacrifice of the Wistar rats.

### Measurement of ciliary beat frequency

Beating ciliated strips were recorded by a high speed video camera (Kodak EktaPro Motion Analyser, Model 1012. San Diego, California, USA) at a rate of 400 frames per second. The camera allowed video sequences to be downloaded at reduced frame rates, allowing ciliary beat frequency to be determined directly by timing a given number of individual ciliary beat cycles. At each time point of the study, ciliary beat frequency was measured at four different areas along each ependymal strip. Only intact, undisrupted, ciliated strips in excess of 100 μm were studied. We have previously shown that measurement of beat frequency using this method is highly reproducible, with the component of variance for intra- and inter-subject variability being only 1 % and 3.8 %, respectively, of the total variation [35].

### Exposure to halothane

A glass bottle was filled with 40 ml of medium 199 with Earle’s salts (pH 7.4: plus penicillin 50 u/ml and streptomycin 50 μg/ml). Ciliated samples were placed in plastic cuvettes within the fluid. Multiple holes were cut in the cuvettes to allow free passage of medium 199. The use of cuvettes allowed the ciliated samples to be suspended in the same region of the bottles for all experiments. The bottle containing a ciliated sample was placed in a heating bath to maintain the temperature of the fluid medium at 37°C. The halothane vaporizer (Flurotec 3 plenum: Cyprane Ltd, Keighley, England) was set to the required level and air passed through it at a flow of 1 l/minute (measured at the distal end of the tubing) taking halothane to the fluid medium surrounding the ciliated samples.

The plastic tubing passed through the lid of the bottle containing medium 199 blowing halothane across the fluid surface. Gas exited the bottle via a separate tube and was vented outside.

Our experimental preparation did not allow frequent measurements to be made during halothane exposure. Attempts were made to measure ciliary beat frequency continually. An incubation system we have developed, which allows continual observation of ciliary beat frequency, was used. Unfortunately, significant evaporation occurred due to air passing over the surface of the chamber, which contains 4 ml of fluid resulting in a significant increase in the concentration of the constituents of medium 199.

Attempts to remove ciliated brain slices from the cuvettes used to perform ciliary beat frequency analysis at regular intervals resulted in significant damage to the fragile ependyma. It was, therefore, decided to make one reading, after three hours exposure to air or halothane.

### Study design

Following incubation of brain slices in medium 199 with Earle’s salts (pH 7.4: plus penicillin 50 u/ml and streptomycin 50 μg/ml), at 37°C for 30 minutes, baseline ependymal ciliary beat frequency was measured, at four different places along an intact ependymal strip. Disrupted ependymal strips or edges less than 100 microns in length were not used.

The brain slice was then transferred to the cuvette, suspended in 40 ml of medium 199 at 37°C, and exposed to the study concentration of halothane for three hours. Control samples were exposed to air at a similar flow for three hours. After three hours, slices were transferred back to the incubation chambers within the heated, humidified environmental chamber surrounding the microscope. When the temperature of the medium 199 bathing the brain slide stabilized at 37°C, ciliary beat frequency measurements were repeated. Samples were kept in the incubation chamber for a further hour, the wash-out phase, and readings were repeated.

In total, 12 brain slices were exposed to 2 % halothane, 8 to 3 % and 8 to 4 %. An attempt was made to study matched controls for all samples exposed to halothane. The experimental set-up was identical for controls. Air rather than halothane was blown across the surface of the fluid medium. The total number of control brain slices was 11 for the 2 % halothane experiment, 6 for the 3 % concentration and 7 for the 4 % concentration.

To determine the exposure of ciliated tissue to halothane, a separate study was conducted to directly measure the equilibration time and levels of halothane in the plastic cuvettes. This was considered important as inhalational anesthetic agents are volatile and adhere to plastics.

Equilibration times were determined for halothane in the fluid immediately adjacent to the brain slices by gas chromatographic analysis (Table 1). Halothane at three different vaporizer settings was delivered to the bottle containing medium 199 and brain slices for three hours. A Flurotec type 3 vaporizer (Flurotec 3 plenum: Cyprane Ltd, Keighley, England was used for all experiments. Aliquots of 200 μl were taken, using a glass syringe, from the area next to where the brain slices were placed at one, two and three hours after exposure to halothane. A further aliquot was taken one hour after the vaporizer was switched off. The aliquots were mixed immediately with n-heptane (100 μl) in glass vials on ice. The vials were sealed to prevent evaporation before analysis. The non-aqueous phase was injected onto a 30-m DB17 megabore column under the following conditions: injection temperature 100°C; oven temperature 90°C; flame ionization detector at 100°C. The amount of anesthetic in each sample was determined from the peak against a standard curve for the agent.

**Table 1:** Percentage halothane in medium 199 bathing ciliated brain slices during the study period

	**Time in minutes**			
	**60**	**120**	**180**	**240**
**% halothane: Study 1**	1.6	1.7	1.8	0.5
**% halothane: Study 2**	2.8	3.5	3.3	0.3
**% halothane: Study 3**	3.0	4.5	4.3	0.4

Halothane was blown across the medium 199 containing the brain slices for 180 minutes. A further reading was taken after a one-hour wash-out period (240 minutes).

These samples were then analyzed by gas chromatography (Perkins Elmer 8410. Waltham, Massachusetts, USA) with a DB-17 column, using helium as the carrier gas and detection by flame ionization that had been standardized for halothane [36].

For halothane standard curves, dilutions of liquid halothane were made with heptane in glass ampoules. Measurements of halothane concentration were carried out in duplicate for each concentration studied.

In the studies to determine the effect of pentobarbitone, ciliated ependymal strips were incubated for 30 minutes in the environmental chamber at a temperature of 37°C. Baseline readings were then made and the surrounding cell culture fluid exchanged for either pentobarbitone sodium or a control solution of medium 199. Readings were repeated at hourly intervals for three hours. Solutions were preheated to 37°C prior to fluid exchange. The effect on ciliary beat frequency of ependymal strips was measured at the following concentrations of pentobarbitone: 25 (n = 10), 50 (n = 9) and 250 μg/ml (n = 8). For each experiment involving addition of pentobarbitone, a control strip of ependyma was studied simultaneously.

### Statistics

An analysis of variance was performed using two factors: halothane concentration and time. The effect of halothane concentration was assessed relative to the variation between slices, while the effect of time was assessed relative to the within-slice variation. A series of contrasts were fitted. For halothane concentrations, each concentration was compared with control. For time, each possible pair of the time points was compared. A separate analysis of variance for each of these combinations of contrasts was performed. For pentobarbitone experiments, the mean of the four replicate measurements of each ependymal slice at each time was analyzed. An unbalanced analysis of variance (using the restricted maximum likelihood procedure: Genstat 5 software) was performed with three fixed factors: 1) pentobarbitone concentration – 25, 50, 250 μg/ml; 2) controls for pentobarbitone experiment; 3) 0, 1, 2, 3 hours. There were two random factors: between slice and within slice components of variance.

## Results

The system established to expose brain slices to halothane took up to two hours to reach a plateau level (Table 1). The mean readings determined by gas chromatography when stable levels were reached after 120 minutes were: 1.8 %, 3.4 % and 4.4 % halothane. Stopping halothane after 180 minutes rapidly reduced the halothane concentration of the solution.

**Figure 1:**
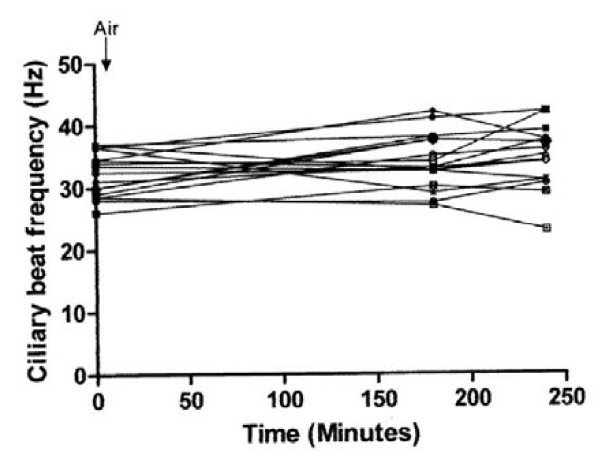
Ciliary beat frequency of ependymal cilia exposed to air.

**Figure 2:**
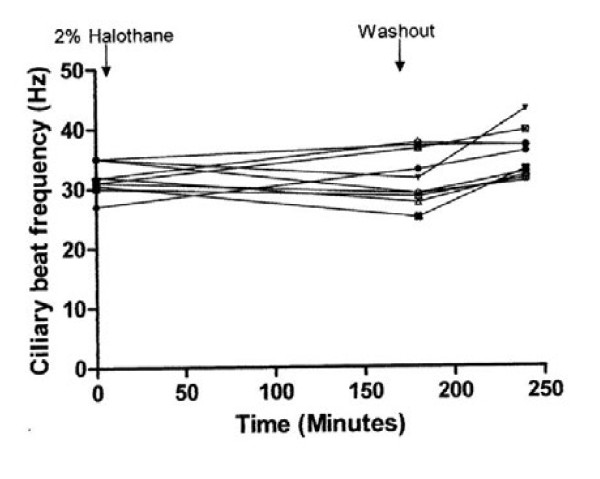
Effect of 1.8 % halothane on ependymal ciliary beat frequency.

**Figure 3:**
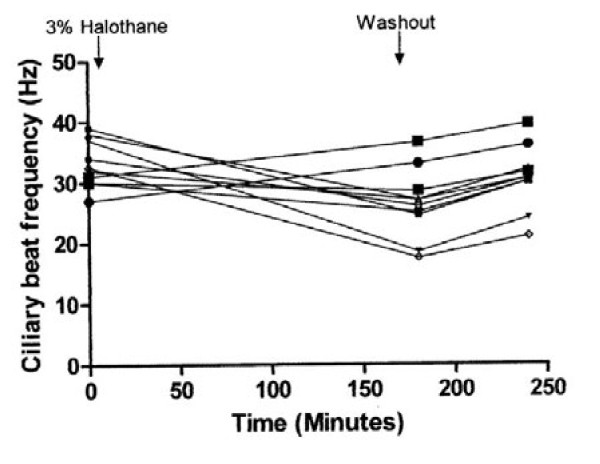
The effect of 3.4 % halothane on ependymal ciliary beat frequency.

**Figure 4:**
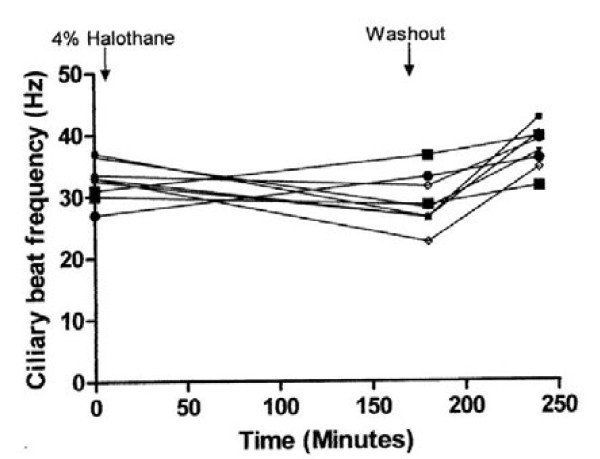
The effect of 4.4 % halothane on ependymal ciliary beat frequency.

The effect of exposure to air controls and 1.8 %, 3.4 % and 4.4 % halothane and the effect of washout period are shown in Figures 1, 2, 3, and 4. There was a highly significant interaction between concentration and time (*P* < 0.001). Compared to controls, significant suppression of ciliary beat frequency occurred after exposure to halothane concentrations of 1.8 % ((*P* = 0.006), 3.4 % (*P* = <0.001), and 4.4 % (*P* = <0.001)) for three hours. This corresponds to a mean increase of 6 % in the ciliary beat frequency of controls compared to a 2 %, 15.5 % and 21.5 % reduction in ciliary beat frequency following exposure to, respectively, 1.8 %, 3.4 % and 4.4 % halothane.

Following a one-hour wash-out period, there was no significant difference between control samples and cilia that had been exposed to 1.8 % (*P* = 0.5) and 3.4 % (*P* = 0.3) halothane. Cilia exposed to 4.4 % halothane had increased in frequency following one hour’s wash-out but were still beating significantly more slowly than cilia from the control group (*P* = <0.001).

At pentobarbitone concentrations of 25 μg/ml and 50 μg/ml, no significant (25 μg/ml; *P* = 0.6: 50 μg/m; *P* = 0.4) effects were found in ciliary beat frequency compared with control samples. At a concentration of 250 μg/ml, however, there was significant (*P* = 0.002) slowing of ependymal ciliary beat frequency (mean (SD) after three hours = 24(8)Hz) compared to control samples (mean (SD) after three hours = 38(9)Hz). Ciliary slowing at a pentobarbitone concentration of 250 μg/ml was not associated with an abnormal beat pattern.

## Discussion

We found the inhalational general anesthetic halothane reversibly inhibited the rate at which ependymal cilia beat while pentobarbitone has no effect on ciliary activity at levels used for anesthesia.

Exposure to halothane concentrations of 1.8 %, 3.4 % and 4.4 % over a three-hour period caused a significant decrease in ependymal ciliary beat frequency. Exposure to the two higher concentrations resulted in a much greater degree of ciliary slowing of up to 21 %. The depressive effects of 1.8 % and 3.4 % halothane were fully reversible when ciliary beat frequency was measured after a wash-out period of one hour. The beat frequency of cilia exposed to 4 % halothane also increased during the wash-out period but was still significantly slower than initial readings. In two slices exposed to 3.4 % and 4 % halothane, there was an increase in ciliary beat frequency. The reason for this is unclear but we have previously observed that an increase in frequency can occur with physical movement of the slices and with an increase in temperature. As temperature was monitored throughout, this is less likely to be the explanation.

Halothane distributes widely throughout the body and significant levels are found in the CSF. Arterial levels of halothane are over 80 % of the inspired concentration and after four hours of exposure to halothane, similar levels are found in the CSF [37]. Following cessation of a halothane anesthetic, CSF halothane decreases significantly more slowly than arterial halothane levels. It is likely, therefore, that halothane administered during anesthesia may induce slowing of ependymal cilia and should not be used in studies which relate to the investigation of ependymal ciliary activity.

It is of interest that brain ependymal cilia from young rats required higher concentrations of halothane to cause ciliary depression than cilia from the respiratory tract of human adults. Such a difference may be due to a number of factors, including differences in species, tissue or age. The effect of inhalational anesthetics, such as halothane, are known to be age-dependent. The potency of inhaled anesthetic agents is expressed in terms of the minimum alveolar concentration (MAC) required to prevent movement in 50 % of subjects in response to a skin incision. Infants have a MAC of 1.2 % which declines gradually to 0.8 in adults [38,39]. The lack of effect on brain ependymal cilia following exposure to 2 % halothane is not in keeping with results of the effect of halothane on respiratory epithelium. Gyi and colleagues [24] found 1.8 % halothane resulted in a 20 % reduction in ciliary beat frequency after two hours exposure. Raphael found a 25 % decrease in frequency at one hour and 40 % after two hours exposure to 2.3 % halothane [25].

Significant variations in the depressive effect of inhalational anesthetics have been found depending on the tissue preparation used. For example, Raphael *et al*. found the depression of ciliary beat frequency using nasal turbinate preparations of 33 %, 25 % and 33 % with three MAC of halothane, enflurane and isofluorane, respectively, differed from that found with nasal brushings, where reductions of 28, 10 and 2 % were seen [25]. Our tissue is more similar to the turbinate preparation in that relatively intact strips of ciliated ependyma attached to neuronal tissue are used. Using rabbit trachea, Lee found reductions in ciliary beat frequency using three MAC of halothane of 22 % [30]. In a study of tracheal mucous transport in dogs using radioactive droplets and scintillation counters, Forbes found a mucous transport rate of 20 % of controls at around three MAC of halothane and enflurane [40,41]. Forbes measured tantalum bronchographic clearance in dogs anesthetized with halothane and found that 1.2 MAC of halothane administered for two hours delayed the clearance of tantalum for more than four hours after the termination of anesthesia [27].

The recovery characteristics following general anesthesia are dependent on the physical properties of the anesthetic agents. Halothane has a high lipid/water solubility coefficient and may take longer to diffuse out from the fat soluble tissues of the preparation at the higher concentration. Raphael and colleagues found the return to baseline values of ciliary beat frequency following exposure to three MAC of halothane, enflurane and isofluorane for one hour took one hour in the cases of enflurane and isofluorane and one and a half hours in the case of halothane [25].

Schettini following measurement of brain water and electrolyte concentration concluded that halothane also induces metabolic brain edema [14]. The movement of CSF by ependymal cilia is in the predicted direction of CSF flow and may serve to clear metabolites and toxins from the brain by improving the diffusion gradient between neuronal tissue and CSF. The relationship between such a role and metabolic brain edema is speculative.

The cellular mechanisms responsible for the activity of halothane remain to be clarified. Halothane and isofluorane alter the tension of isolated cerebrovascular smooth muscle, a response that is further modulated by the CO_2_ tension [42]. The vasodilatory action of halothane is thought by some to involve nitric oxide (NO) [43] but by others to be independent of it [44]. Indeed, halothane reduces the vasodilatory effect of NO [45], an effect that ought to attenuate vasodilatation. It is generally agreed, however, that halothane depletes intracellular (sarcoplasmic) CA^2+^ stores [46,47]. Another interesting finding is that volatile anesthetics produce hyperpolarization in snail neurones [48].

Sodium pentobarbital is a short acting barbiturate used in humans for pre-operative sedation and in the treatment of seizures. In veterinary medicine it is used as an anesthetic. Reassuringly, we found sodium pentobarbital at concentrations likely to produce deep coma (25 μg/ml) and at levels capable of inducing a flat EEG (50 μg/ml) [49] had no effect on brain ependymal ciliary beat frequency. Although significant slowing was seen following incubation with a concentration of 250 μg/ml, cilia were still beating at half of their initial rate and had a normal beat pattern. Highly perfused, relatively slow, volume tissues, such as the brain, equilibrate rapidly with the high early concentrations of barbiturates, such as pentobarbitone, in the flood, resulting in induction of anesthesia [50]. Drug levels then decrease quickly as the drug redistributes throughout the body. Our study suggests that the use of pentobarbitone as an anesthetic for experiments investigating the effect of ependymal ciliary movement *in vivo* is justified. The gamma-amino butyric acid (GABA) receptor complex is thought to be the most likely site of barbiturate action. Barbiturates both enhance and mimic the action of GABA [51]. By binding to their receptors, barbiturates decrease the rate of dissociation of GABA from its receptor and increases the duration of GABA activated chloride ion channel openings. At higher concentrations, barbiturates directly activate chloride channels, even in the absence of GABA. Although pentobarbitone levels in the CSF have not been measured, the only concentration of pentobarbitone causing an effect on ciliary function was at a level considered lethal to humans. The mechanism behind ciliary response at this concentration is uncertain.

## Conclusions

We have shown halothane reversibly inhibits the rate at which ependymal cilia beat while pentobarbitone has no effect on ciliary activity at levels used for anesthesia. The slowing of respiratory cilia by inhalational anesthetics may lead to mucous retention predisposing to post-operative chest infection. The clinical effect of acute slowing of brain ependymal cilia is unknown. It is yet to be determined whether slowing of ependymal ciliary beat frequency is responsible for some of the secondary central nervous system effects of these agents. The lack of effect of pentobarbitone on ependymal ciliary function at levels used during anesthesia is reassuring.

## Abbreviations

CSF, cerebrospinal fluid; GABA, gamma-amino butyric acid; MAC, minimum alveolar concentration; NO, nitrous oxide.

## Competing interests

The authors declare that they have no competing interests.

## Authors’ contributions

CO’C designed the study, established the methods, analyzed the results and wrote the manuscript. KS measured ciliary function and halothane levels. All authors read and approved the final manuscript.
